# The Global Influenza Surveillance and Response System—65 years of building trust and sharing and a role model for global health security

**DOI:** 10.1111/irv.12548

**Published:** 2018-06-25

**Authors:** Wenqing Zhang, John Wood

**Affiliations:** ^1^ Global Influenza Programme World Health Organization Geneva Switzerland; ^2^ Retired affiliate of the National Institute for Biological Standards and Control London UK

Among all pathogens of public health concern, influenza virus is unique and somewhat notorious for its ability to constantly evolve and to rapidly spread; its potential for catastrophic impact; and its ubiquitous presence in wild and domestic animals. The only way to address such a serious challenge is by timely and effective global collaboration.

One such collaboration began 65 years ago: the WHO Global Influenza Surveillance Network (GISN), just 4 years after the WHO constitution had came into force.^1^ Beginning with only 26 laboratories in 1952 to the current situation where there are now 152 institutions in 113 countries, the Network has been vital in protecting the world from influenza. When the Network began to expand its functions and capacity to reflect its role in response mechanisms and upon the adoption of the Pandemic Influenza Preparedness Framework for the Sharing of Influenza Viruses and Access to Vaccines and Other Benefits^2^ by the World Health Assembly in 2011, the Network was renamed the “Global Influenza Surveillance and Response System” (GISRS). By way of celebrating the success of GISRS over the past 65 years, representatives from nearly 100 countries including those from 88 National Influenza Centres attended a special WHO meeting at WHO Headquarters, Geneva, Switzerland, on 17‐19 July 2017. In this issue of *Influenza and Other Respiratory Viruses*, there are 2 reviews by Dr Thedi Ziegler, Awandha Mamahit and Dr Nancy Cox and by Drs Alan Hay and John McCauley describing the historical contributions and the current status of GISRS and then speculating over what the future may hold for GISRS. These 2 reviews are drawn from some of the presentations made at the WHO meeting.

As described in the reviews, GISRS plays a vital role in protection against seasonal influenza, pandemic influenza and influenza viruses with pandemic potential. In the Report of the Review Committee on the Functioning of the International Health Regulations (2005) in relation to Pandemic (H1N1) 2009,^3^ which was considered by the World Health Assembly in 2011, GISN was highly commended and included a statement that it was “the first time that a worldwide laboratory initiative was well‐coordinated for an extended period of time.”

The GISRS Network today exists as a tribute to several generations of scientists throughout the world who have dedicated themselves to influenza surveillance and response. They have shared data, resources and ideas and collaborated in a public‐spirited manner, which is not often seen in science and public health today. It is also a tribute to many governments who have committed their political, financial and administrative support to the Network over the years. GISRS, with its high level of performance and demonstrated success, is a role model for effective global health security, and its features have been incorporated in surveillance and response networks for other disease agents.

In contrast to the usual retirement age of 65 for a person, GISRS is in the prime of life at 65. In the modern and high‐tech era, the world is not immune to influenza. This year we commemorate the centenary of 1918 Spanish Flu, which gives us a timely reminder of the devastating consequences of pandemic influenza. With all the complexities and competing priorities of the modern world, let GISRS be preserved and strengthened for the next 65 years.
